# Angioimmunoblastic T‐cell lymphoma: a rare subtype of peripheral T‐cell lymphoma

**DOI:** 10.1002/ccr3.1388

**Published:** 2018-02-10

**Authors:** Manoj Ponadka Rai, Prabhjot Singh Bedi, Edwin B Marinas, Nazia Naz S. Khan

**Affiliations:** ^1^ Michigan State University/Sparrow hospital B301, 788 Service Road East Lansing Michigan 48824; ^2^ UPMC East 2775 Mosside Blvd Monroeville Pennsylvania 15146; ^3^ Sparrow Hospital 1215 E Michigan Ave Lansing Michigan 48912

**Keywords:** Angioimmunoblastic T‐cell lymphoma, autologous transplantation, Immunostaining (CD2, CD3, CD4, CD10, CXCL‐13, PD1, BCL‐6), nonhodgkin's lymphoma, T‐cell lymphoma

## Abstract

Angioimmunoblastic T‐cell lymphoma (AITL) is a rare form of NHL and usually presents in the late stage due to the atypical laboratory findings. Immunohistochemistry of the lymph node in AITL is characterized by positive CD2, CD3, CD4, CD10, CXCL‐13, PD1 often BCL‐6 and CD20 positive. Meshworks of follicular dendritic cells are seen outside follicles with CD21 and CD23 stains. EBV can be often positive as well. Autologous transplantation should be offered in the first remission as poor outcome is reported with anthracycline‐containing regimens.

## Case

**Figure 1:**
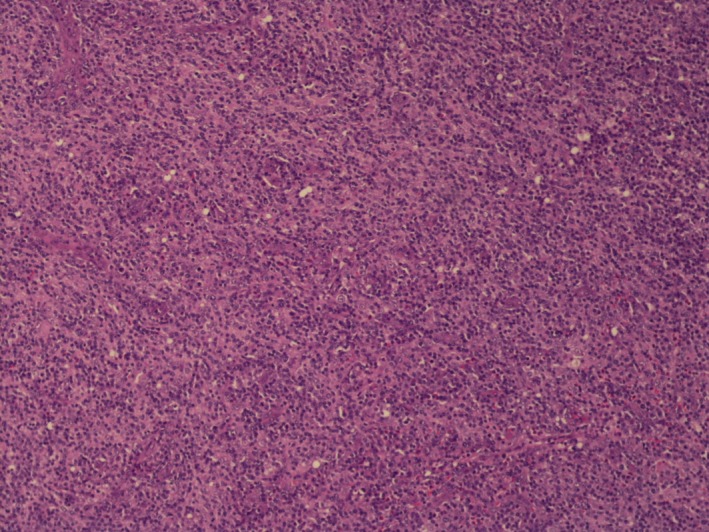
Lymph node biopsy showing effaced architecture on 10× resolution.

**Figure 2:**
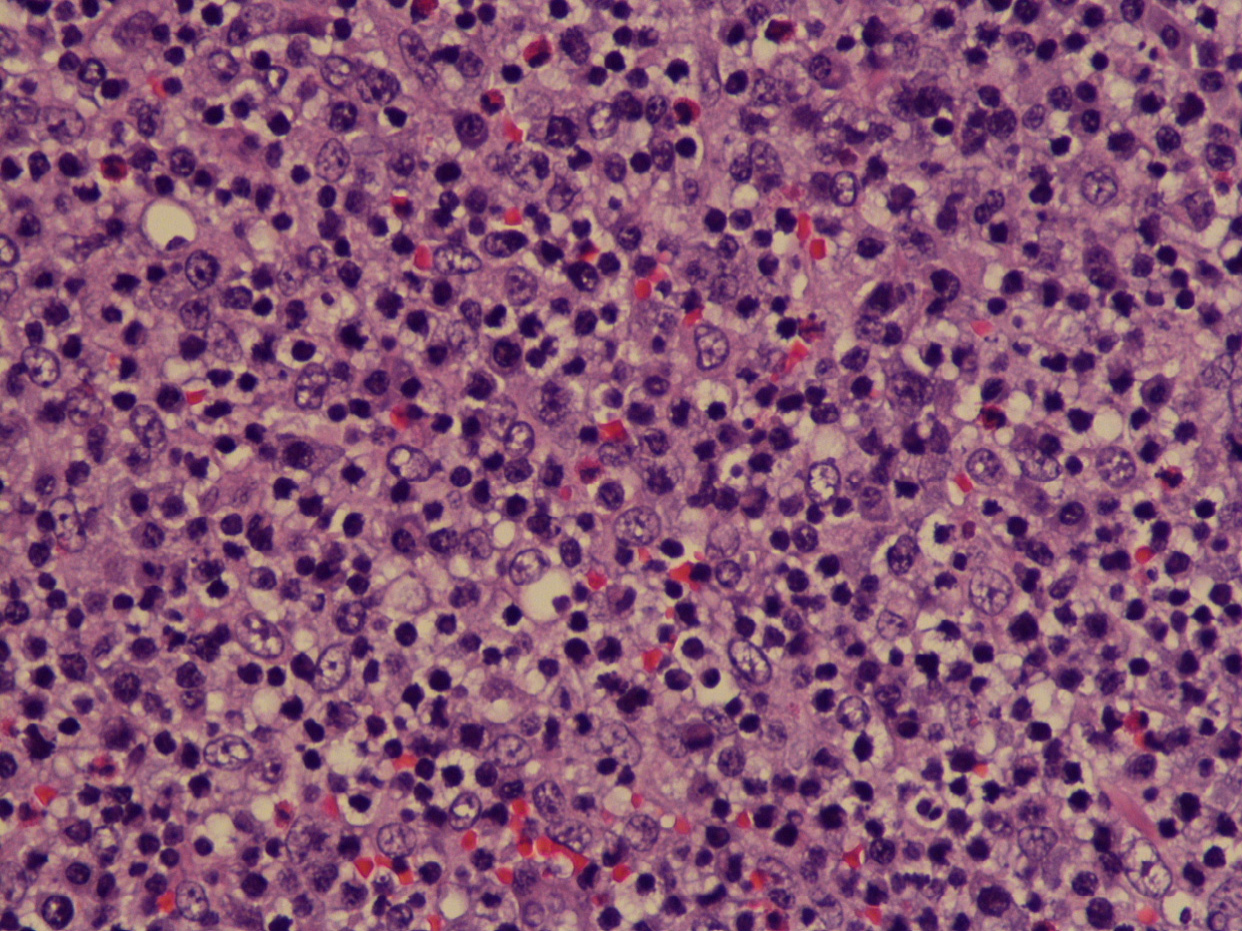
Lymph node biopsy showing scattered large lymphoid cells on 40× resolution.

**Figure 3:**
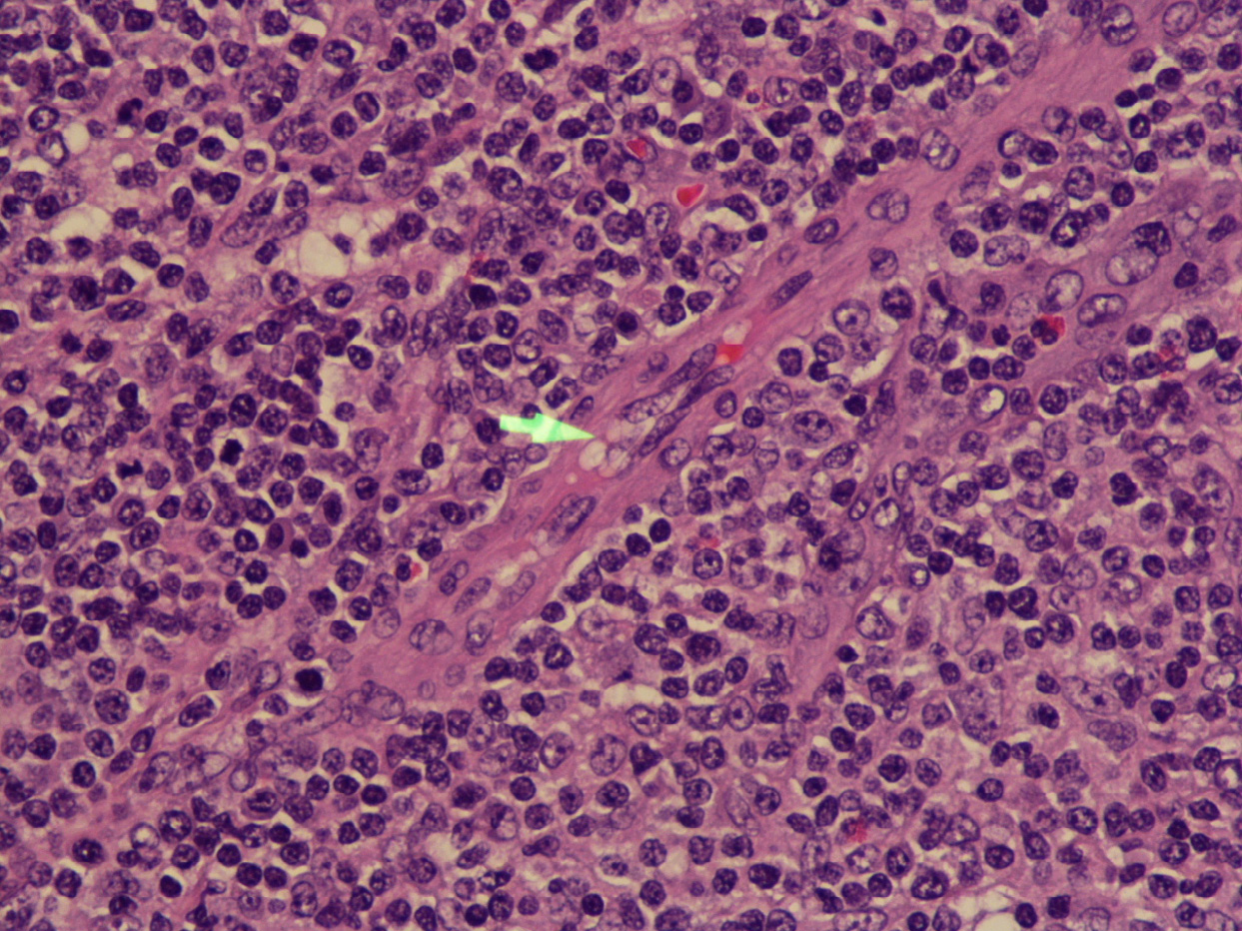
Lymph node biopsy showing vascular proliferation on 40× resolution.

**Figure 4:**
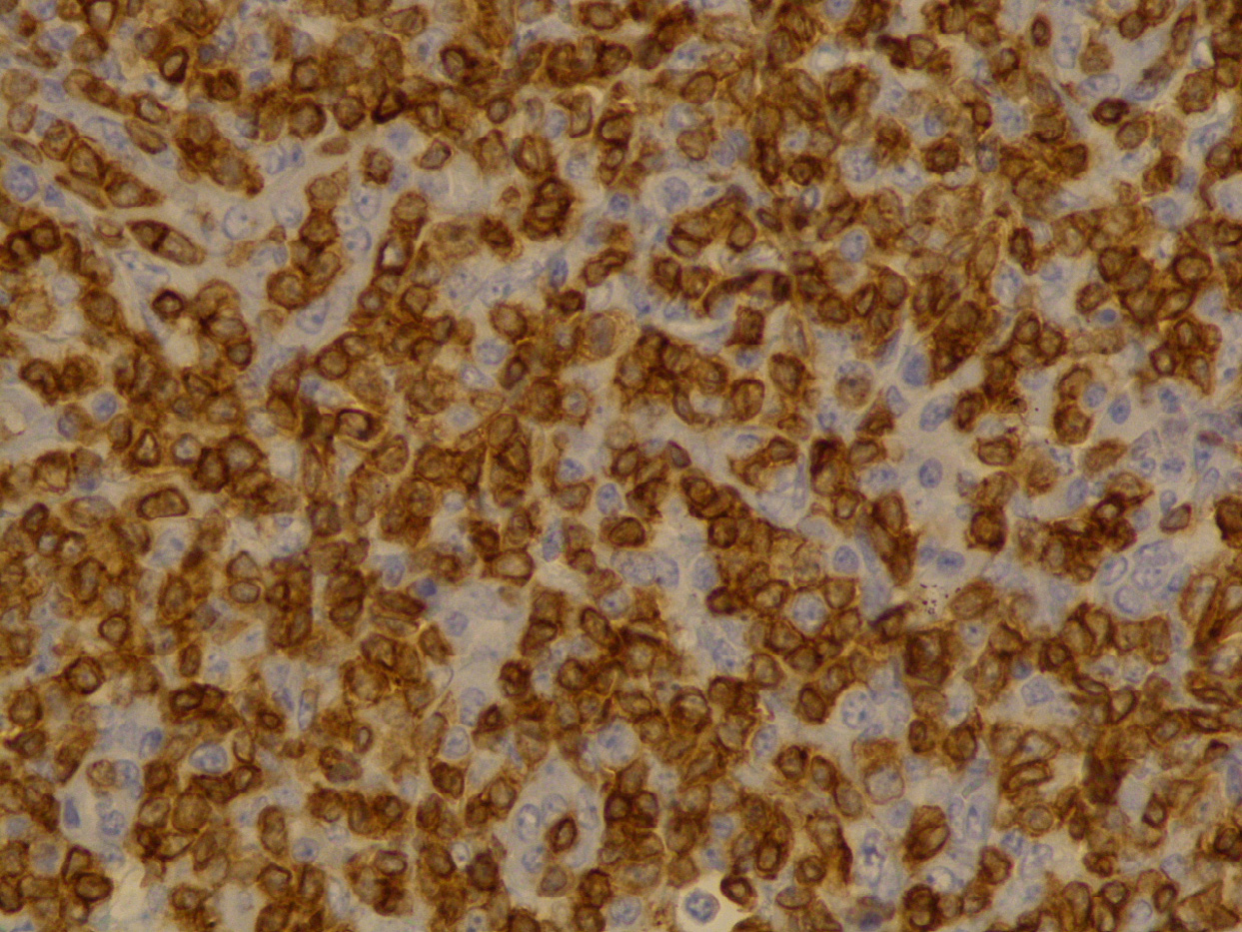
CD3 (T lymphocyte marker) immunostain 40× resolution.

This is a 68‐year‐old female who presented with diffuse myalgias, loss of appetite, and 4.5 kg unintentional weight loss over 1 month. It was associated with periodic fevers up to 38.3°C and night sweats. On physical examination, she was found to have right‐sided cervical lymph node enlargement and generalized abdominal tenderness. Computed tomography of the abdomen and pelvis revealed diffused small lymph nodes and splenomegaly. Our differentials were broad, and it included viral lymphadenitis such as infectious mononucleosis, leukemia, lymphoma, tuberculosis, and hypersensitivity syndrome. Mononucleosis screening, Cytomegalovirus, and Epstein–Barr virus IgM were negative. Tuberculosis was low on the differentials as the patient did not have any history of exposure or travel. She was not on any medications known to cause hypersensitivity syndrome. Biopsy of the lymph nodes showed effaced architecture (Fig. 1) and expanded paracortical areas by an atypical lymphoid infiltrate composed predominantly of small‐ to medium‐sized cells with irregular nuclei, inconspicuous nucleoli, and moderately abundant cytoplasm. There were also few scattered larger lymphoid cells (Fig. 2), some plasma cells, histiocytes, and eosinophils. There was a prominent vascular proliferation (Fig. 3) in the paracortical areas. Immunophenotyping by flow cytometry showed no evidence of a monoclonal B lymphocyte population and no evidence of aberrant T‐cell antigen expression. Immunohistochemical stains showed CD20, PAX5, and CD79A highlight, expanded B‐cell nodules, and scattered B‐cells interspersed among the T‐cells. Lymphoma cells in the paracortical areas were positive for CD2, CD3 (Fig. 4), CD5, CD43, TIA‐1, and BCL2 (weak, partial). CD4 positive cells mildly predominated over CD8 positive cells. CD10, BCL6, and PD‐1 highlight increased the number of positive cells, in an abnormal pattern, mainly in and around the B‐cell nodules. CD21 and CD23 showed expanded follicular dendritic cell meshworks. CD30 highlighted larger scattered cells. CD15 stained granulocytes/eosinophils but was otherwise negative. Epstein–Barr encoding region (EBER) in situ hybridization was positive in scattered cells. CD138 stains scattered plasma cells, which were polyclonal by kappa and lambda immunohistochemical stains. Proliferative index, highlighted by Ki67, was 40–50%. Molecular studies were positive for rearrangements of T‐cell receptor gamma (TRG) and beta (HRB). In summary, morphologic, immunophenotypic, and molecular studies were consistent with angioimmunoblastic T‐cell lymphoma. Angioimmunoblastic T‐cell lymphoma (AITL) accounts for 1.2% nonhodgkin's lymphoma and about 18% of T‐cell lymphomas 1. The mean age of presentation is 64.5 years, and no patient was found younger than 30 years 2. 60% of cases were found to have bone marrow infiltration at the time of diagnosis. Most patients were present with polyadenopathy and general symptoms, and more than one‐third had immune cytopenia, neuropathy, polyarthritis, or vasculitis. Uncharacteristic laboratory and autoimmune findings often show or mask the diagnosis, and thus, it is usually diagnosed at an advanced stage 3. Staining of the lymph node is positive for CD2, CD3, CD4, CD10, CXCL‐13, PD1, and often BCL‐6 4. They are frequently CD20 positive, often EBV positive as well 5. Outside the follicles and usually around vessels, stains for CD21 and CD23 typically show meshworks of follicular dendritic cells. In 30% of patients, T‐cell receptor gene rearrangements are negative, and immunoglobulin gene rearrangement can be found in about 10% of AITL patients. Poor outcome is reported with anthracycline‐containing regimens; therefore, whenever possible, autologous transplantation in the first remission should be offered. Innovative induction strategies (CHOP + biologic agent) should be designed to enhance response quality, facilitate transplantation, and prolong survival.

## Conflict of Interest

None declared.

## Authorship

MPR: wrote the case description. PB and NNSK: contributed to the description on AITL and the key clinical message. EBM: provided the description of the pathology images.
